# The Right Kind of Evidence—Integrating, Measuring, and Making It Count in Health Equity Research

**DOI:** 10.1007/s11524-012-9738-y

**Published:** 2012-07-07

**Authors:** Patricia J. Martens

**Affiliations:** Manitoba Centre for Health Policy, University of Manitoba, Winnipeg, Canada

**Keywords:** Integrated knowledge translation, Health inequity measures, Longitudinal analysis of socioeconomic gaps, Lorenz curve, Relative versus absolute risk, Manitoba Centre for Health Policy, The Need To Know Team

## Abstract

As health equity researchers, we need to produce research that is useful, policy-relevant, able to be understood and applied, and uses integrated knowledge translation (KT) approaches. The Manitoba Centre for Health Policy and its history of working with provincial government as well as regional health authorities is used as a case study of integrated KT. Whether or not health equity research “takes the day” around the decision-making table may be out of our realm, but as scientists, we need to ensure that it is around the table, and that it is understood and told in a narrative way. However, our conventional research metrics can sometimes get in the way of practicality and clear understanding. The use of relative rates, relative risks, or odds ratios can actually be detrimental to furthering political action. In the policy realm, showing the rates by socioeconomic group and trends in those rates, as well as incorporating information on absolute differences, may be better understood intuitively when discussing inequity. Health equity research matters, and it particularly matters to policy-makers and planners at the top levels of decision-making. We need to ensure that our messages are based on strong evidence, presented in ways that do not undermine the message itself, and incorporating integrated KT models to ensure rapid uptake and application in the real world.

For purposes of an expert forum entitled, “Power, Politics, and the Use of Health Equity Research” held in Toronto on February 17–18, 2011, a panel was formed to discuss what does, what could, and what should count as research use. We were asked to address three questions: (a) what kind of research “impact” do we want and can we expect from urban health and health equity research?, (b) conventional knowledge translation (KT) metrics and social determinants of health research: Are we set up to fail?, and (c) are we finally producing the right kind of evidence to advance urban health and health equity? My stance on this question focuses on the experience of working in an integrated KT environment at the Manitoba Centre for Health Policy and on what this has taught me about displaying inequity data appropriately to decision-makers.

## The Right Kind of Evidence: Research Collaborations and Integrated KT Models

### The Manitoba Centre for Health Policy as a Case Study of Integrated KT

I began working as a research scientist at the Manitoba Centre for Health Policy (MCHP) in 1999 and became its director in 2004. This is a unit in the Department of Community Health Sciences, Faculty of Medicine at the University of Manitoba, and has been in existence for over two decades (since 1990). MCHP has a unique stance in terms of an academic research center—it has an ongoing grant relationship with Manitoba Health which brings in approximately half of the funding (the other half being research grants from the Canadian Institutes of Health Research (CIHR) and other national and provincial granting agencies). The MCHP/Manitoba Health grant supports three endeavors: (a) ensuring that the repository of administrative claims data upon which our research is based is maintained and enhanced; (b) ensuring knowledge translation of research; and (b) determining five research projects a year, in consensus discussions. The relationship with Manitoba Health has been ongoing since its start, and the need for policy relevant yet academically based research has proven its worth throughout various political eras. Historical analyses and recommendations for replicating the establishment of a centre like MCHP are discussed in a special supplement of the *Healthcare Policy* journal,^1^ written in conjunction with MCHP's 20th anniversary celebrations in 2010.

The Director of MCHP, along with the government liaison person (the Executive Director of Health Information Management Branch of Manitoba Health), compiles 30 to 40 ideas from various sources throughout the year—ideas from researchers, clinicians, regional health authorities, from within government itself and ideas that may have been on a previous year's list but did not make it to the top five. Each project must be feasible from the perspective of MCHP and deemed highly relevant or timely in terms of the government's long-term need for research on which to base policy or programs. Four of the projects are directly determined through discussions with Manitoba Health's Executive Management Committee and Minister of Health and the fifth through discussions with the Healthy Child Committee of Cabinet. Chaired by the Minister of Children and Youth Opportunities, this committee includes ministers from nine departments of government (including health, healthy living, education, family services, justice, and others) that influence the health of children.^2^ Once the five topics are determined annually, MCHP's Director and Associate Director of Research designate a lead scientist and a support team (research coordinator, data analyst, support staff, and co-investigators) for each of the projects, and this group meets weekly until the completion of the project (which usually takes around 2 years). The lead scientist also forms a suitable advisory group which meets two to three times a year throughout the project. This group consists of topic experts from a variety of perspectives - government, regional health authorities (RHAs), clinicians, program planners, and other research scientists locally and nationally.

MCHP's research projects funded through Manitoba Health are grounded in the reality of decision-making while maintaining the highest standards of academic research (see^1^,^2^) in how questions will be answered, how results will be interpreted, and when the results will be released. The public release is usually preceded by briefings to key stakeholders (for example, the Minister of Health) in the weeks leading up, while the final report is being edited and printed. As well, the scientists publish their findings in peer-reviewed journals. This type of environment within a university is highly unusual for the traditional academic. By definition, the very stance of MCHP is integrated KT.^3^,^4^ User involvement (i.e., in this case, provincial government involvement) is critical in the dialogue of determining which five questions will be researched each year and in ensuring that the results reach the top levels of decision-making.

### The Need to Know Team and the MCHP Annual Workshops

Since 2001, MCHP has also been involved in an integrated KT relationship with the RHAs of Manitoba. *The Need to Know* Team (directed by myself and co-directed by Dr. Randy Fransoo) was established through a CIHR grant in 2001 and maintained through various CIHR grants ever since (including my current CIHR/PHAC Applied Public Health Chair). This is a group of research scientists and graduate students from MCHP, high-level decision-makers and planners from each of the 11 Manitoba RHAs and from Manitoba Health. There are many published articles describing this Team, but the essence is that the team focuses on new knowledge creation of direct relevance to RHA decision-making, capacity building amongst all team members, and dissemination/application of research findings at the RHA level and beyond.^5^–^8^ We meet three times a year, for 2 days at a time. In our decade together, we have produced major research reports and publications and have presented at conferences and invited workshops. RHAs use the results of our collaborative research heavily in their mandated 5-year planning documents, since they must show an evidence-based approach to planning in order to ensure their ongoing funding from the province.

*The Need to Know* Team is heavily involved throughout the research process itself, with critiquing and helping put context onto results, helping in the writing of publications, using the research at ground-level planning within the RHAs, and contributing to cross-Canada dissemination of results through conferences. But MCHP has also had a history of interactive learning sessions with the RHAs and with Manitoba Health, through what we call our Annual Health Care Days—one for the non-Winnipeg RHAs, one for the Winnipeg RHA, and one for Manitoba Health. These days are very similar in structure, with one MCHP research report highlighted in a plenary session in the morning, followed by roundtable discussions of the meaning and implications of the findings. The plenary speaker does not focus on the results of the report but rather how to read the report (for example, what does the statistical testing mean, what indicators are in the report, and what analyses have been done). At the Rural and Northern Health Care Day, most of the RHA CEOs and board members, as well as Medical Officers of Health, VPs of planning, managers of quality assurance, frontline workers, and interested parties are around that table. The table is facilitated by *The Need to Know* Team members from that RHA along with an MCHP scientist to help clarify the research where necessary. The roundtable discussion leaders frame the discussions around the results of the report, finding out the “What, So What, Now What” stories in the results. As well, discussions focus around levels of intervention necessary to change things—what downstream (curative), midstream (education or preventive), and upstream (policy or environmental) approaches are needed or what further information is required. These discussions will, of course, be very RHA-specific due to the differing contexts and geographical implications. Working in the environment of MCHP and its collaborative research agenda with provincial and regional health planners and policy-makers, its integrated KT strategies, and its various forums for disseminating information helps shape my way of looking at what kind of evidence truly makes a difference.

## The Right Kind of Evidence—Uptake by Decision-Makers

In the use of family services, education, and health data within MCHP, several research scientists have done internationally recognized work on describing socioeconomic gaps.^9^ The repository held at MCHP contains administrative claims databases for virtually the entire population of Manitoba, including data across both health and social services. The repository databases are all geocoded and longitudinal, de-identified yet linkable at the person level using maximum privacy and confidentiality policies and procedures.^1^,^2^ The advantage of linking information such as children in care, children in families on social assistance, and children of teen mothers to outcomes in educational attainment (both at the start of schooling and at the high school graduation endpoint) enabled the researchers to speak directly to the relevant deputy ministers of government departments, school board members, the Business Council, the United Way, and many other groups interested in child outcomes. According to Roos et al.^9^, there are four success factors in getting research into action: (a) continually pursue opportunities to dialogue with the relevant decision-makers, building up understanding and trust; (b) use local data organized by some measure of socioeconomic status (SES), so that the message of inequity is strong and not easy to ignore; (c) focus on the outcome measure of educational attainment—this helps develop a strong and broad constituency for taking action and also attracts the business community into the discussions; and (d) show research that the poor outcomes in the lower SES groups are not inevitable, but rather can be overcome through appropriate action. Their findings have resulted in such action as the implementation of the Community Schools Initiative, a low-income community education strategy, the creation of a social policy agenda in the Business Council, the impetus to keep high school students in school, the All Aboard Strategy to help youth make the transition from foster care to independent living, and the adding in of more subsidized day care and nursery school spaces in the lowest SES areas of Winnipeg.

In an article entitled “Tales from the Tectonic Plate,”^8^ we reflected on lessons learned both at MCHP and by similar researchers across Canada when interacting with policy-makers. We observed that relationships, sustainable funding (especially with some funding coming from decision-makers themselves), and making research relevant were critical to uptake of research. Interestingly, we have learned many of the same lessons from a decade of researcher–knowledge user collaboration in *The Need to Know* Team. In working with these decision-makers, we have learned that relationships and personal factors are critical—building trust and confidence between the researchers and the users of this research is essential, but takes time, commitment, and ongoing resources.^6^ We have also learned that creation and sustainability of a collaborative research environment requires in-person ongoing contact, as well as shared vocabulary and common understandings of research and its application. This does not come without funding—ongoing sustainable funding is critical to making integrated KT a reality.

## The Right Kind of Evidence—What Counts around the Decision-Making Table?

Are we producing the right kind of evidence to advance health and health equity? And what kind of research impact do we want and can we expect from health and health equity research? The right kind of evidence is probably a moot point if we work in an integrated KT mode—“right” by research standards means the best possible approaches to answer the questions in the most valid and reliable way, and right by decision-maker standards means a research project which answers something relevant and of high importance.

As research scientists, we all hope for an impact that is something visible, tangible, and measureable. But that may take years, or it may not happen at all. I have come to believe that my impact as a health services and population health research scientist is to produce appropriate and relevant research (usually using integrated KT models), but also to ensure that this research is “around the decision-making tables.” This means that part of my KT job is to ensure its dissemination, but also ensure that I have done my best to help people understand and contextualize the research. If people are given the opportunity to look for “evidence-based stories” in the data that make sense to them, are true to the research, and are contextualized in their own experiences, then these people will remember the stories—and more important, bring these evidence-based stories to the decision-making table. As a research scientist, I need to be pragmatic enough to realize that research will not always take the day in the decision—sometimes politics, economics, values, and beliefs will take the day.^10^ But my job is to make sure that research is in the discussion (i.e., at the table) and will be there in their back pockets (what I call the back pocket mindset) in the future when the topic arises again.^8^

Sometimes we can measure the direct impact of our research in the policy realm, and sometimes, it is more nebulous. Using MCHP as a case study, Lewis et al.^10^ identified several layers of impact for a health services and population health research centre. It is often the case that the road from awareness of evidence to widespread implementation takes a very long time to travel. On the other hand, the very act of awareness, understanding, and discussions may eventually change cultural norms or ways of thinking, so these are important features relating to measuring research impact on decision-making. On the downside, these aspects are difficult to quantify or even to attribute directly to one specific study.^10^

## The Right Kind of Evidence—Using the Right Measures for Quantitative Health Equity Research Findings

### Relative Risks, Relative Rates, and Odds Ratios—Risky Business in Health Equity Presentations

MCHP recently completed a report on health inequities,^11^ to explore socioeconomic gaps in urban and rural Manitoba and to determine whether these gaps were widening, narrowing, or staying similar over time. This was in support of an initiative by Manitoba Health and the Chief Public Health Officer to reduce provincial inequity. How this drives political and public will for change remains to be seen. However, it gave us the opportunity to quantify both the magnitude of the inequities and the direction of future intervention—should it be only universal or universal plus targeted? This was influenced by the Marmot Review^12^ and the idea of proportionate universalism. The lesson learned in doing this MCHP report, from a research point of view, was in how to measure gaps and what data best speak in a decision-making realm.

**FIGURE 1.**
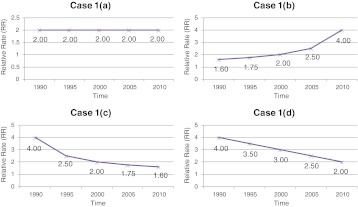
Graphs of RR over time, comparing low SES to high SES groups' disease rates: What message would a decision-maker obtain from each graph (**a** to **d**)?.

**FIGURE 2.**
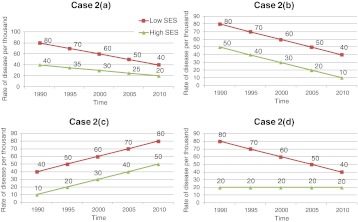
Graphs of actual rates of disease of the highest and lowest SES groups over time: What message would a decision-maker obtain from each of these graphs (**a** to **d**)?.

As a simple exercise in the take-home message of health inequity graphs for decision-makers, try to the do following exercise. Look at all of the graphs in Figure 1. Write down the “take home” message that a decision-maker would receive for each graph (a to d), in terms of health equity progress (or not) over time. Do the same for all four graphs in Figure 2.

After completing this exercise, compare what you have written down for each parallel graph (Figure 1a compared to Figure 2a, then Figure 1b compared to Figure 2b, etc.). Table 1 indicates possible messages that you may have written. Each of the parallel graphs is derived from the same underlying data, but displayed either as relative rates (RR) over time (comparing the lowest SES group to the highest SES group) or as the real rates of both the low and high SES groups over time. Interestingly, the only scenario where decision-makers would intuitively get the same take home message is in the last set of graphs (Figures 1d and 2d). However, researchers by nature tend to rely on relative risks (RR) or odds ratios (OR), simply because that is what the statistical tests often generate. This may not necessarily translate into an intuitive message for decision-makers.

**Table 1:** Potential interpretation of Figure 1 and 2 graphs by a decision-maker

Graphs from the figures	Interpretation by the decision-maker	How the two graphs differ in interpretation by the decision-maker
Figure 1a	Inequity has stayed similar over time, at an RR of 2—we are making no progress in health inequity reduction	The conclusion of the decision-maker differs markedly between the two graph displays—one is interpreted as no progress, the other as substantial progress
Figure 2a	In both the low and high SES groups, disease rates are going down, with even more rapid improvement in the low (so that we are seeing a shrinking gap, i.e., risk difference)—we are making real progress both in overall health improvements and in shrinking the gap	

Figure 1b	Inequity has increased over time and appears to be exponentially getting worse—we are in trouble	The conclusion of the decision-maker differs markedly between the two graph displays—one is interpreted as deterioration and increasing health inequity, the other as progress in overall health but similar inequity over time
Figure 2b	In both the low and high SES groups, disease rates are going down at a steady rate, with similar improvements for both groups—we are making real progress in overall health improvements, despite the fact that the real difference in disease burden has remained the same between the two groups	

Figure 1c	Inequity has shrunk over time, with a dramatic change originally and less progress more recently—we are on the right track, but the progress is slowing down	The conclusion of the decision-maker differs markedly between the two graph displays —one is interpreted as making progress, the other as deteriorating health in both groups with no progress in reducing health inequity
Figure 2c	In both the low and high SES groups, disease rates are going up at a steady rate, with similar deterioration in health for both groups—we have a problem, with increasing disease rates, but at least the real difference between the groups is staying similar and not widening the health inequity	

Figure 1d	Inequity continues to shrink over time—we are definitely on the right track, so continue the course	The conclusion of the decision-maker is similar for both graphs—improvements in decreasing health inequity
Figure 2d	There have been tremendous improvements in the health of the low SES group over time, whereas the high SES group has stayed similar over time—we see great progress in increasing the health of the lowest SES group, and the health inequities have shrunk, so we are on the right track (except that the high SES group seems “stalled out” in health improvements)	

Which is the right message for health inequity researchers to use in their dissemination of research findings? Obviously, the graphs showing the real rates over time by SES group, as well as the RR graphs over time, are both mathematically correct. But what is the appropriate information from both a researcher and a decision-maker perspective? The only situation in which RRs and real rates gave a similar take home message was Figures 1d and 2d, where the highest SES group's actual disease rate did not change over time. This type of finding rarely happens in real-life data.

I would argue that Figure 2 types of graphs are more appropriate for decision-makers. Why? RR can be very deceiving as the two real rates (high and low SES groups) change over time. No matter how the population's health improves over time, RR types of graphs like in Figure 1 will mostly give depressing news. As the high SES group's disease rate approaches 0, even if the lowest SES group's disease rate is a very small number, the RR will go up exponentially and approach infinity! Is that clinically or practically meaningful? No. You can see this effect happening in Figures 1b and 2b, where the RR graph tells a bad news story for the decision-maker, yet the real rate graph tells a very encouraging story. So, for both researchers and policy makers, setting health equity targets using relative rates may actually be highly detrimental to advancing political or social will to address the problem of reducing inequity. As researchers, we rely too heavily on reporting RR and OR and not enough on reporting the true rates (or rate differences in actual terms, not relative terms). In clinical epidemiology, researchers avoid using relative risks for comparing the outcomes of clinical treatments due to the potential misunderstandings of these. Rather, actual rate differences or number needed to treat (NNT) is used to avoid the pitfall of overstating an effect. Now, we need to learn the same lesson in population health, especially in health equity research.

**FIGURE 3.**
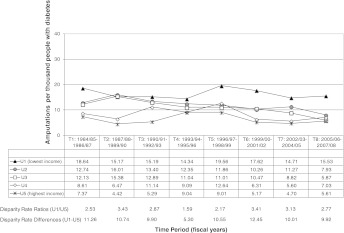
Rate of amputation due to diabetes over time by Manitoba urban income quintile, showing actual rates, rate ratios, rate differences, and comparisons (from^11^, with permission). Adjusted by (fiscal years 2005/06 - 2007/08) age and sex, for annual rate per thousand residents with diabetes (aged 19 and older) who had an amputation.

So what do we, as health equity researchers, do about this? When we produce graphs, we should give as much information as possible to as many audiences as possible, but the main focus of graphs we share with decision-makers should give a picture of the real rates over time by SES grouping (or whatever groupings are used). A graph derived from the report by Martens et al.^12^ is shown in Figure 3. The term “urban” refers to the combination of Winnipeg and Brandon, the two major urban centers within the province of Manitoba. This is an example of a graph that shows both the real rates over time, as well as rate ratios and rate differences of the lowest (U1) and highest (U5) income quintile groups. The focal point, however, is the actual rates over time, to give a more intuitive look that is of interest to the policy-makers.

### Promoting the Use of Lorenz Curves to Display Health Inequity

In the health inequity report by Martens et al.^11^, we also explored the use of Lorenz curves as a means of describing inequities. These proved to be very useful for decision-makers. Lorenz curves were a great way to visualize inequity, changing inequities over time, and rural/urban inequity differences.

**FIGURE 4.**
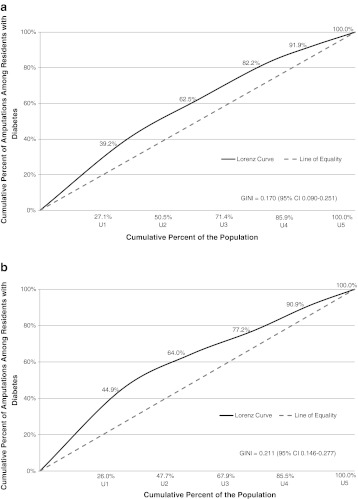
Adjusted Lorenz curves (adjusted for age and sex to fiscal years 2005/06-2007/08) for amputation due to diabetes at two time periods, showing the cumulative proportion of amputations in people with diabetes with increasing income quintile group for urban Manitoba.^11^ **a** Lorenz curve for fiscal years 1984/85-1986/87 **b** Lorenz curve for fiscal years 2005/06-2007/08.

Figure 4 shows two Lorenz curves for lower limb amputation due to diabetes, from two time periods, for urban Manitoba. Basically, the bigger the bend, the greater the disparity (inequity)—this is a highly visual representation of disparity that decision-makers intuitively understand and can use to compare “gaps” over a variety of indicators. In the first time period, 27 % of the people with diabetes were in the lowest urban SES group (i.e., the lowest income quintile group, U1), but this group had 39 % of the amputations in the population. In the last time period, things have become worse, with the 26 % of people with diabetes in that bottom quintile having close to 45 % of the amputations. The Gini coefficient (a way to measure the degree of bend) showed a statistically significant increase in inequity over time (0.170 to 0.211, *p* < .05). The line of equity (the dashed line in the curves) represents the situation in which each SES group would have an “equal” portion of disease comparable to the proportion of the population in that group (for example, if there were 21 % of the population in the lowest SES group, they would have 21 % of the disease, and so on). So, if the lowest SES group has a disproportionate amount of illness compared to their portion of the population, the curve bends away from the dashed line. The bigger the inequity, the greater will be the bend in the curve and the greater the value of the Gini coefficient.

We need to produce quantitative evidence of inequity, and we need to display this in a way which is not only reliable and valid, but useable and fair from a decision-maker's point of view. This is critical to the uptake and use of research for social change.

## Conclusions

Our panel was asked to address questions on what kind of research impact do we want and can we expect from urban health/health equity research, what metrics should we use, and what is the right kind of evidence to produce in order to advance urban health and health equity. So, in conclusion, my viewpoint is that we need to produce research that is useful, policy-relevant, and able to be understood and applied, and that this research should be done in an integrated KT collaboration. Whether or not this research “takes the day” around the decision-making table may be out of our realm, but as scientists we need to ensure that it is around the table, and that it is understood and told in a narrative way.

Our conventional research metrics can sometimes get in the way of practicality. So, we need to be especially cautious about using relative risks or odds ratios and possibly rely more heavily in the policy realm upon the true rate, or trends in rates, and in absolute differences when discussing inequity. In the scientific stance, we never get to a place where we can rest on our laurels. The very nature of science is dynamic, with an ever changing understanding of what is a right answer, what is a correct approach, and what is a truly meaningful finding. So although in some ways we can say we may be producing good evidence, there is always room in the scientific world of policy-relevant research to do even better. Health equity research matters, and it particularly matters to policy-makers and planners at the top levels of decision-making. We need to ensure that our messages are based on strong evidence, presented in ways that do not undermine the message itself, and incorporated integrated KT models to ensure rapid uptake and application in the real world.

## Footnotes

1 The *Healthcare Policy* special issue, “Going for the Gold: Celebrating 20 Years of Experience in Population-Based Research in Manitoba and Beyond,” can be downloaded at http://www.umanitoba.ca/faculties/medicine/units/community_health_sciences/departmental_units/mchp/media/Healthcare_Policy_vol6_MCHP.pdf

2 The Healthy Child Committee of Cabinet is described at the following website, accessed March 7, 2011:
http://www.gov.mb.ca/healthychild/about/welcome.html
